# *Drosophila melanogaster* As a Model Organism to Study RNA Toxicity of Repeat Expansion-Associated Neurodegenerative and Neuromuscular Diseases

**DOI:** 10.3389/fncel.2017.00070

**Published:** 2017-03-21

**Authors:** Alex C. Koon, Ho Yin Edwin Chan

**Affiliations:** ^1^Laboratory of Drosophila ResearchHong Kong, Hong Kong; ^2^Biochemistry ProgramHong Kong, Hong Kong; ^3^Cell and Molecular Biology ProgramHong Kong, Hong Kong; ^4^Molecular Biotechnology Program, Faculty of Science, School of Life SciencesHong Kong, Hong Kong; ^5^School of Life Sciences, Gerald Choa Neuroscience Centre, The Chinese University of Hong KongHong Kong, Hong Kong

**Keywords:** polyglutamine disease, Huntington's disease, C9orf72, amyotrophic lateral sclerosis (ALS), frontotemporal dementia (FTD), myotonic dystrophy, fragile X, spinocerebellar ataxia

## Abstract

For nearly a century, the fruit fly, *Drosophila melanogaster*, has proven to be a valuable tool in our understanding of fundamental biological processes, and has empowered our discoveries, particularly in the field of neuroscience. In recent years, *Drosophila* has emerged as a model organism for human neurodegenerative and neuromuscular disorders. In this review, we highlight a number of recent studies that utilized the *Drosophila* model to study repeat-expansion associated diseases (READs), such as polyglutamine diseases, fragile X-associated tremor/ataxia syndrome (FXTAS), myotonic dystrophy type 1 (DM1) and type 2 (DM2), and C9ORF72-associated amyotrophic lateral sclerosis/frontotemporal dementia (C9-ALS/FTD). Discoveries regarding the possible mechanisms of RNA toxicity will be focused here. These studies demonstrate *Drosophila* as an excellent *in vivo* model system that can reveal novel mechanistic insights into human disorders, providing the foundation for translational research and therapeutic development.

## Introduction

For nearly two decades, the fruit fly *Drosophila melanogaster* has been utilized as a model organism to study a number of human neurodegenerative and neuromuscular diseases (Chan and Bonini, 2000; Muqit and Feany, 2002; Zoghbi and Botas, 2002; Sang and Jackson, 2005; Marsh and Thompson, 2006; Yu and Bonini, 2011; Jaiswal et al., 2012; McGurk et al., 2015). The fruit fly offers multiple advantages for the investigation of the molecular mechanisms of diseases. Having short life cycle, high offspring numbers, low costs for maintenance, simple yet powerful genetic manipulations, the availability of mutants, and other genetic tools are only some of the many attractive features of *Drosophila* as a model system. *Drosophila* was introduced into scientific research over a 100 years ago, and quickly became an invaluable tool that empowers our discoveries and understanding of a wide range of biological processes, such as embryogenesis, neural development, synaptic plasticity, and even complex behaviors such as decision-making and learning and memory (Bellen et al., 2010; Spindler and Hartenstein, 2010; Harris and Littleton, 2015). Sequencing of the *Drosophila* and the human genomes revealed remarkably high similarities between the fly and humans (Adams et al., 2000; Rubin, 2000), with ~75% of the genes implicated in human genetic disorders having at least one homolog in *Drosophila* (Rubin, 2000; Reiter et al., 2001). Most importantly, despite the obvious anatomic divergence between the fly and humans, the fundamental aspects of cell biology are highly conserved, including the regulation of gene expression, synaptogenesis, cell proliferation, cell differentiation, cell signaling, and cell death. Many genes and pathways that are being studied in mammals were originally identified in *Drosophila*. For instance, the mammalian *Wnt* gene was originally identified as *wingless* in *Drosophila* with its mutation resulting in flies having no wings (Sharma and Chopra, 1976). *Wnt*/*wingless* is now widely known for its involvement in a broad variety of conserved cellular processes and human diseases (Korkut and Budnik, 2009).

## The advantages of using *Drosophila* for studying human disease-associated genes

Comparing with mammals, *Drosophila* has much simpler genetics. The fruit fly has only four pairs of homologous chromosomes, as compared to 20 in mice and 23 in humans. Flies also have a much simpler nervous system, with ~200,000 neurons in the fly brain comparing to ~100 billion neurons in humans. Despite its simplicity, the fly nervous system is comprehensive, allowing the fly to perform a wide variety of behaviors, such as feeding, walking, climbing, courtship, and communication. Fruit flies are even capable of being trained with fear/reward conditioning paradigms to examine complex biological processes like learning and memory. These features of *Drosophila* make it an ideal organism for modeling complex disorders because it can be used to model a specific subset of phenotypes associated with a particular disease, which will simplify the analysis.

A wide range of genetic manipulation techniques have been developed in *Drosophila* which are impractical to be implemented in mammals. For instance, the availability of *P*-elements mutants in libraries is certainly an advantage that is exclusive to the *Drosophila* community. Originally, *P*-elements are stretches of transposable DNA consisting of inverted repeats surrounding a transposase gene to allow hopping of the mobile element on chromosomes (Rubin and Spradling, 1982; O'Hare and Rubin, 1983). *Drosophila* researchers exploited its mobility, and designed *P*-elements without the transposase but with reporter genes. By pairing up this modified *P*-element with an inducible transposase, the *P*-element can then be randomly inserted into the genome to disrupt the transcription of downstream genes. With this technique, libraries of “*P*-element mutants” for tenths of thousands of genes are constructed (Cooley et al., 1988; Bier et al., 1989; Spradling et al., 1999; Bellen et al., 2004). Researchers can now conveniently access the information of these mutants from FlyBase (http://flybase.org), and order them from resource centers such as the Bloomington *Drosophila* Stock Center. In addition, due to the nature that the mobilization of the *P*-elements are typically imprecise, these fly lines provide a means to generate excision mutants (Hummel and Klämbt, 2008). Lastly, the introduction of flipase recombination targets into *P*-elements have provided the option of generating precise chromosomal deletions between two *P*-elements (Hummel and Klämbt, 2008). The development of these genetic tools have greatly enhanced the process of genome editing in *Drosophila*.

Another popular technique used in *Drosophila* research is the GAL4/UAS binary transgene overexpression system, which is perhaps one of the most versatile expression system ever developed in *Drosophila* (Brand and Perrimon, 1993). GAL4 is a yeast transcription factor that drives the expression of a transgene downstream of an *Upstream Activation Sequence* (*UAS*). Usually, a GAL4 fly line expresses GAL4 under the control of a cell- or tissue-specific promoter. This can be achieved by enhancer-trap screens, or the fusion of identified promoters with the GAL4 gene and subsequently microinjecting the constructs into fly embryos to produce specific GAL4 driver lines (Duffy, 2002). For the operon, a human disease gene of interest is usually subcloned into an expression construct containing UAS, which is then also microinjected into fly embryos to produce transgenic fly lines. The UAS operon lines will be crossed to GAL4 fly lines, resulting in the overexpression of the human disease gene of interest in the desired cells/tissues (Duffy, 2002). If the expression of pathological human genes in the fly successfully yields an abnormal phenotype, such as degeneration in the photoreceptor neurons of the fly eye, genetic screens, and pharmacological screens can then be performed to search for genetic modifiers and small molecules that may impact the etiology of the corresponding human disorders.

One of the most prominent aspects of the fly model is its capacity to conduct genetic screens to identify novel molecular components of pathological pathways. Mice models of human diseases are absolutely vital to advancing our understanding of the diseases, but nevertheless with limitations. In *Drosophila*, the classical forward genetic screens can be performed using mutagens such as ethyl methane sulfonate to generate mutants (St Johnston, 2002; Yamamoto et al., 2014). Since this approach is unbiased, it allows the identification of novel genes from the entire genome, which would otherwise be extremely difficult to perform in mouse models. Due to having a short life cycle, the simplicity of having only four pairs of homologous chromosomes and the availability of balancer chromosomes for the suppression of recombination, the isolation of mutations from *Drosophila* is much easier than that from a mouse. Apart from forward genetic screens, tools for high throughput reverse genetic screens are also available. Genetic tools for RNAi-mediated knockdown are easily accessible from stock centers such as the Vienna *Drosophila* Resource Center and Harvard Transgenic RNAi Project, making it possible to conveniently perform reverse genetic screens in the fly model (Dietzl et al., 2007).

External features of *Drosophila* makes it suitable for performing large scale screens of genes and pharmacologicals. Bristles, wing veins and the compound eye are all external structures of the organism that can be affected by genetic or cellular changes, and can easily be observed and scored under a stereomicroscope (Yamamoto et al., 2014). The compound eye is mostly composed of photoreceptor neurons, and is commonly used as a model to monitor neurodegeneration, allowing the identification of novel genetic modifiers and drugs in screens (St Johnston, 2002). Using the GAL4/UAS system, toxic RNAs or toxic proteins that commonly occurs in human neurodegenerative and neuromuscular diseases can be expressed in the fly eye, which often results in a rough-eye phenotype. Since the eye is not required for viability, this allows effective screening of genes or pharmacologicals that can alleviate or enhance the degeneration. Most compounds can be simply mixed into standard *Drosophila* food to be consumed by larvae and adult flies. Alternatively, compounds can be injected into adult flies as well if a high, acute dosage is needed in the adult stage. The identification of novel genetic modifiers or drugs may reveal the physiological role of disease-related genes and promote our understanding of the disease pathology (Zoghbi and Botas, 2002; Hirth, 2010; Pandey and Nichols, 2011; McGurk et al., 2015). Thus, *Drosophila* serves as an important platform for the discoveries of new components of pathological pathways and the development of pioneer therapeutic approaches.

## The limitations of modeling human diseases in the fruit fly

Although the use of classical genetic screens of *Drosophila* provides an unbiased approach to gain insights into human degenerative diseases, fly mutations may not be precise representations of human disease mutations. This is because the classical fly mutants from forward genetic screens are typically loss-of-function alleles. Yet in human diseases, mutations can have complex presentations, including both loss-of-function of the wild type allele and gain-of-function of the mutant allele. Most of the times, this gain-of-function effect can still be modeled in *Drosophila* by overexpressing the mutant protein using the GAL4/UAS system (St Johnston, 2002). But for some diseases which the loss-of-function of the wild type protein plays a major role in the disease etiology, if there are no *Drosophila* orthologs of the corresponding human disease-associated genes, then perhaps the fly is not a suitable model for the particular disease.

Furthermore, while the GAL4/UAS system is an extremely effective and versatile system, it is after all an overexpression system that may give rise to the typical caveats of overexpression studies. The magnitude of overexpression in a model can be tremendously different from the clinical situation (Floresco et al., 2005). Sometimes, even overexpressing a wild type version of a gene may cause a disease phenotype (Prelich, 2012). In fact, excess GAL4 protein in the *Drosophila* eye may lead to degeneration (Kramer and Staveley, 2003). Hence, it is paramount for *Drosophila* researchers to set up appropriate controls to ensure that the phenotypes observed are not simply overexpression artifacts.

Lastly, it is a concern of whether the fly models can faithfully recapitulate the selective neuronal death and associated phenotypes of human neurological disorders. Although the *Drosophila* photoreceptor degeneration provides a convenient readout, it mostly reflects generic neurotoxicity instead of selective neurotoxicity that is disease-specific. Thus, it is important to verify relevant phenotypes using other systems that are more specific to the particular types of disease. For instance, for myotonic dystrophy, it is crucial to verify any degeneration phenotype in a neuromuscular system, like the *Drosophila* or mouse neuromuscular junction (de Haro et al., 2006; Panaite et al., 2013).

Despite all the limitations, *Drosophila* has so far proven to be a valid and useful tool in unraveling the etiology of human diseases and identifying compounds that can improve symptoms and modify the course of human diseases. Success examples include the rescuing of disease-like phenotypes in fly models of fragile X syndrome (Chang et al., 2008), prolonged survival of dopaminergic neurons in fly models of Parkinson's disease (Auluck et al., 2005; Faust et al., 2009) and lifespan extension in fly models of Alzheimer's disease (Rajendran et al., 2008).

## Repeat expansion-associated neurodegenerative and neuromuscular diseases

As early as 1918, unstable nucleotide (microsatellite) repeat expansion had already been identified as a mutational mechanism underlying several human disease (Fleischer, 1918). Originally, only trinucleotide repeats were associated with human diseases (Fu et al., 1991; La Spada et al., 1991). But over the years, other disease-causing expandable microsatellite repeats were gradually discovered including unstable tetra-, penta-, and hexa-nucleotide repeats (Mirkin, 2007). Remarkably, the majority of the repeat expansion-associated diseases (READs) manifest with neurological and neuromuscular symptoms. Until today, more than 20 neurodegenerative and neuromuscular disorders have been linked with unstable nucleotide repeat expansions in the human genome (Pearson et al., 2005; Brouwer et al., 2009; Todd and Paulson, 2010; Polak et al., 2013).

**Figure 1:**
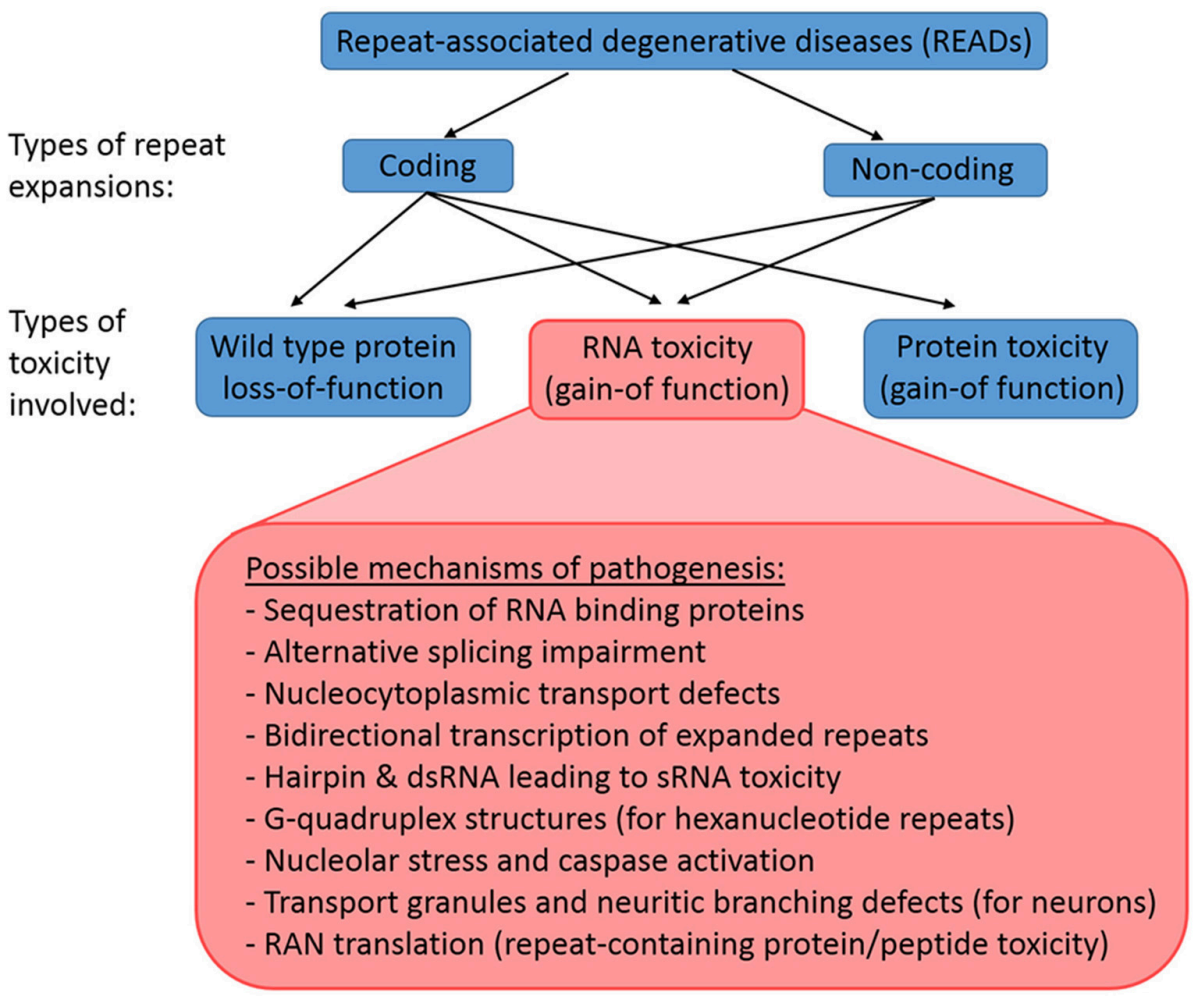
**Classification of READs and their types of toxicity**. READs can be classified into coding and non-coding. A repeat expansion that occur in the coding region of an affected gene may result in the loss-of-function of the wild type proteins, the gain-of-function of toxic RNAs, and the gain-of-function of toxic proteins. In contrast, a repeat expansion in the non-coding region of an affected gene would not lead to immediate protein toxicity, but could involve the loss-of-function of the wild type proteins and the gain-of-function of toxic RNAs. However, RAN translation may still occur in non-coding READs, which results in the production of toxic repeat-containing proteins/peptides.

Repeat expansions can be coding or non-coding, and the underlying mechanism of a READ may involve (1) loss-of-function of wild-type protein, (2) gain-of-function of toxic RNAs, or (3) gain-of-function of toxic proteins (Figure 1). Coding expansion disorders usually result from smaller repeat copies (few to tens of copies) that manifest in exon coding regions of genes. This group of READs includes mostly polyglutamine (polyQ) diseases like Huntington's disease (HD) and several forms of spinocerebellar ataxia (SCA), which typically involves the translation of the gene products together with the repeat expansions into toxic proteins with gain-of-function (La Spada et al., 1991; MacDonald et al., 1993; Orr et al., 1993; Kawaguchi et al., 1994; Imbert et al., 1996; Pulst et al., 1996; Sanpei et al., 1996; David et al., 1997; Zhuchenko et al., 1997; Nakamura et al., 2001). In contrast, the non-coding expansions disorders typically involve larger expansions (from ~100 to 1000 copies), which reside in the non-coding regions of genes, such as promoters, introns or untranslated regions (UTRs). This groups includes fragile X-associated tremor/ataxia syndrome (FXTAS), Friedrich's ataxia, C9-ALS/FTD, myotonic dystrophy types 1 and 2 (DM1 and DM2) and SCA8 (Brook et al., 1992; Montermini et al., 1997; Jin et al., 2003; Mutsuddi et al., 2004; DeJesus-Hernandez et al., 2011; Renton et al., 2011). Non-coding expansions usually results in repeat-containing transcripts with gain-of-function RNA toxicity. However, even non-coding expansions can still be translated into toxic peptides by repeat-associated non-ATG translation (Zu et al., 2011). Thus, the pathologies of READs are complex, as both coding and non-coding repeat expansions may involve a combination of mechanisms, including wild-type proteins loss-of-function, toxic RNA gain-of-function, and toxic protein gain-of-function.

## RNA gain-of-function toxicty in reads and its pathogenic mechanisms

**Figure 2:**
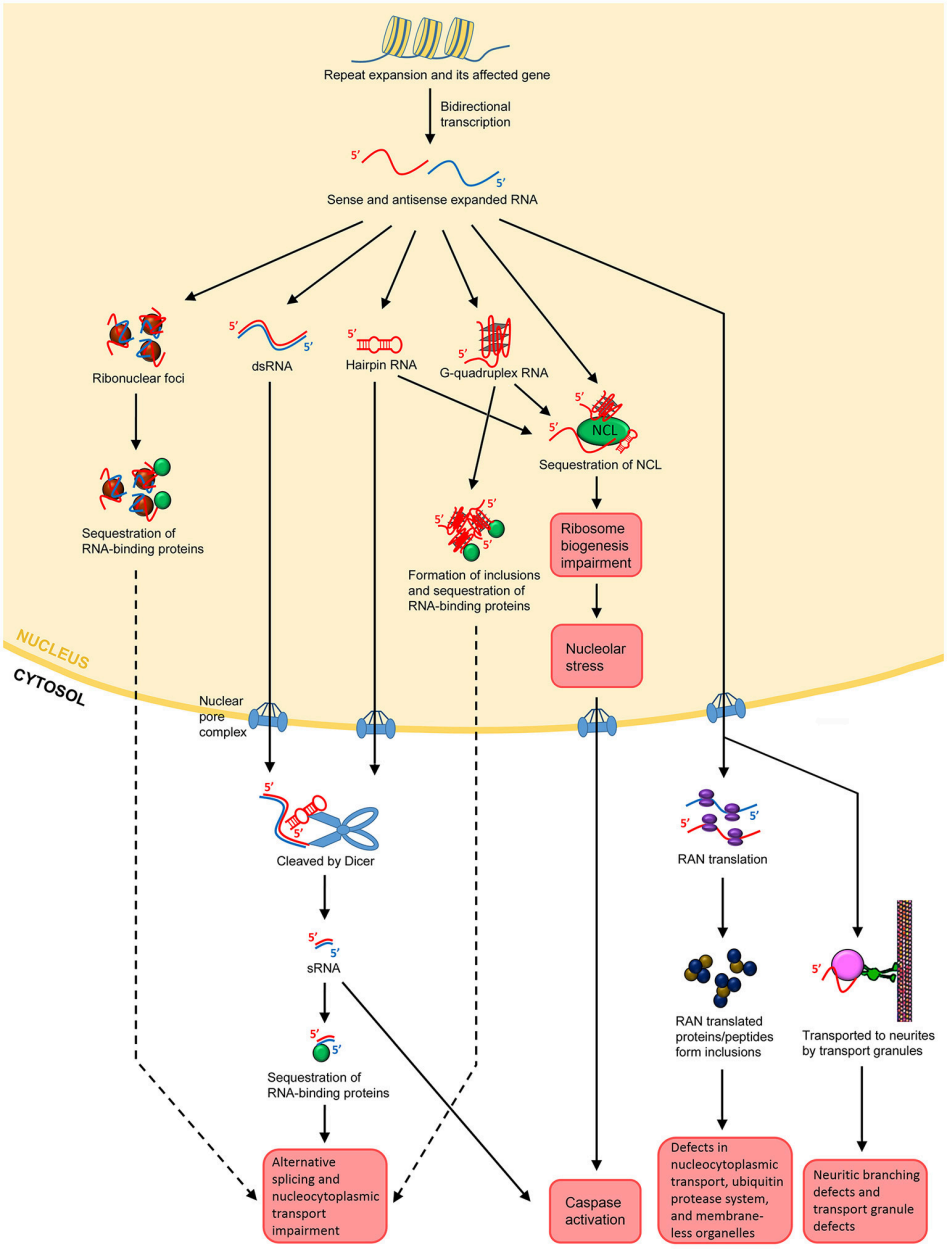
**Possible mechanisms of RNA toxicity in repeat expansion-associated diseases**. Repeat expansion can occur in coding or non-coding regions of affected genes. Bidirectional transcription results in both sense and antisense transcripts containing the repeats. Both sense and antisense expanded RNAs can form ribonuclear foci. The foci may sequester RNA-binding proteins, such as MBNL1 in the case of DM1, leading to impairment of the alternative splicing and nucleocytoplasmic transport machineries. The sense and antisense ssRNAs can anneal to form dsRNAs. ssRNAs can also form hairpin structures by themselves, which are degradation resistant. Both dsRNAs and hairpin-forming RNAs may export the nucleus and be cleaved into sRNAs by Dicer. The sRNAs can then sequester RNA-binding proteins, leading to splicing and nucleocytoplasmic transport impairment. For hexanucleotide repeats such as GGGCC in C9-ALS/FTD, the ssRNA may form G-quadruplex structures. These structures facilitate DNA/RNA hybrid and impede transcription. They also form inclusions, leading to the sequestration of RNA-binding proteins as well. Repeat-containing transcripts, whether in hairpin or G-quadruplex structures, can bind to NCL, inducing nucleolar stress and caspase activation. Expanded transcripts that escape the nucleus associate themselves with ribosomes, leading to RAN translation. RAN translational products may form inclusions, which disrupts nucleocytoplasmic transport, impedes the ubiquitin protease system and impairs the assembly, dynamics, and functions of membrane-less organelles such as the nucleolar and stress granules. In neurons, the expanded transcripts may also be actively transported to neurites, resulting in neuritic branching, and transport granule defects. It is postulated that the local translational machinery may also be disrupted.

The concept that RNA itself acts as a major toxic species in neurodegenerative/neuromuscular disorders was first described for DM1. DM1 is the most common form of muscular dystrophy in adults, caused by an expanded CTG repeat in the 3′UTR of the *dystrophia myotonica protein kinase* (*DMPK*) gene (Brook et al., 1992; Fu et al., 1992; Mahadevan et al., 1992). Ribonuclear foci were found to be forming in the nuclei of the affected cells, and these foci contain the RNA transcripts from the CTG repeat expansion (Taneja et al., 1995). In brief, the expanded repeats localized at the foci in turn sequester endogenous RNA binding proteins, impairing the alternatively splicing mechanisms and leading to cellular toxicity and degeneration (Figure 2; Renoux and Todd, 2012; Belzil et al., 2013; Fiszer and Krzyzosiak, 2013; Nalavade et al., 2013). This is the “sequestration hypothesis” or “alternative splicing impairment model,” which is one of the most supported mechanisms of the emerging “RNA toxicity model.” The ribonuclear foci are now a hallmark for most of the READs associated with non-coding repeats (Wojciechowska and Krzyzosiak, 2011). Some even coined the term “RNAopathies” for these disorders due to the clear involvement of RNA toxicity. Noted that, despite the consensus that the formation of foci is a feature of pathogenesis, their role in pathogenesis is still unclear. The original belief was that they could be toxic since they harbor the toxic RNA transcripts. Yet, accumulating evidence from DM1, DM2 and C9-ALS/FTD studies now suggests that the foci *per se*, do not appear to be toxic, since cells with foci do not necessarily degenerate (Houseley et al., 2005; Yu et al., 2011, 2015; Mizielinska et al., 2014; Tran et al., 2015). The fact that cells with the same amount of foci could very well be alive or dying indicating that toxicity is not proportional to foci numbers (Mizielinska et al., 2014; Tran et al., 2015). Thus, ribonuclear foci could be neutral or even beneficial, at least in the beginning phase of the disease progression.

An alternative mechanism of RNA toxicity is bidirectional transcription. The original discovery for bidirectional transcription came from a study on SCA8. SCA8 is a type of spinocerebellar ataxia arises from a CTG expansion in the 3′ end of a non-protein coding RNA, *Ataxin-8 Opposite Strand* (*ATXN8OS*; Koob et al., 1999). Its pathogenic mechanism was thought to be similar to DM1 since both disorders involves a CTG repeat. However, the antisense transcript, *Ataxin-8*, has a short cryptic open reading frame that results in CAG repeats in the glutamine reading frame (Moseley et al., 2006). Moreover, nuclear polyQ inclusions were found, indicating that both toxic RNA and toxic proteins gain-of-function were being produced and could be involved in the pathogenesis of SCA8 (Moseley et al., 2006). Bidirectional transcription was found in models of DM1, FXTAS and SCA7, suggesting that it is a common mechanism of RNA toxicity in READs (Cho et al., 2005; Ladd et al., 2007; Sopher et al., 2011).

A third mechanism of RNA toxicity is nucleolar stress and nucleocytoplasmic transport defects. Nucleolar stress is defined as the cellular pathways used by the nucleolus to communicate with cytosolic compartments in order to trigger apoptosis (Boulon et al., 2010; Lindenboim et al., 2011). Hence, nucleolar stress is a conserved and effective mechanism to eliminate ribosome biogenesis-defective cells. It has been reported that CAG repeat expansion in polyglutamine diseases interacts directly with nucleolin (NCL), a nucleolar protein that regulates rRNA transcription and ribosome biogenesis (Tsoi et al., 2012). The CAG repeat-induced impairment of ribosome biogenesis in turn increases the concentration of p53 in mitochondria, disrupts the interaction between anti- and pro-apoptotic factors such as Bcl-xl and Bak, causes cytochrome c release and caspase activation, and eventually leads to cell death (Tsoi et al., 2012). In polyQ diseases, the fact that CAG repeats-containing RNAs are retained in the nucleus indicates defective nucleocytoplasmic transport of the mutant transcripts (Tsoi et al., 2011). It was reported that CAG repeats can directly bind to the U2 small nuclear ribonucleoprotein auxiliary factor 65 (U2AF65), the fly homolog of human U2AF50 that is involved in the nuclear export of mRNA. U2AF65 is capable of forming a complex with the nuclear export receptor NXF1 to regulate nucleocytoplasmic transport of RNAs (Tsoi et al., 2011). Thus, the downregulation of U2AF65 due to sequestration by CAG repeats results in the nuclear accumulation of RNAs (Tsoi et al., 2011). Furthermore, the researchers observed a neurodevelopmental downregulation of U2AF65 protein levels in a mouse model of HD, suggesting that the reduction in U2AF65 levels causes nuclear accumulation of expanded CAG-containing transcripts in HD. Apart from polyQ diseases, nucleocytoplasmic transport defects have also been demonstrated in models of C9-ALS/FTD (Freibaum et al., 2015; Zhang et al., 2015). These studies demonstrate the involvement of the nucleolar stress and nucleocytoplasmic transport defects in the RNA toxicity-mediated pathogenesis of READs.

A forth mechanism of RNA toxicity is mediated by hairpin-forming single stranded RNAs (ssRNAs) and double-stranded RNAs (dsRNAs) containing the expanded repeats. Both of them would eventually lead to the generating of toxic repeat-containing small RNAs (sRNAs), which is the true culprit of this mechanism of RNA toxicity in READs. It is known that hairpin structures can be formed by single stranded CAG repeats in polyQ diseases and CGG repeats in FXTAS (Sobczak et al., 2003, 2010). Complex secondary structures like hairpin loops can significantly alter the transcripts' processing, transport, translation, and interactions with RNA binding proteins. Hairpin structures also strongly protects the RNAs from attacks by nucleases, rendering them more resistant to degradation (Sobczak et al., 2010). Hence, the hairpin structure itself already conveys a type of toxicity. However, the more severe consequences come from the fact that it facilitates the production of sRNAs via the RNAi machinery. Dicer is an RNase III family ribonuclease that cleaves double-stranded precursor RNAs to generate sRNAs, and is a key enzyme involved in RNAi (Bernstein et al., 2001). Since expanded ssRNAs in READs form double-stranded hairpin structures, they are valid substrates of Dicer, and will be cleaved into sRNAs. Even though these repeat-containing sRNAs cannot form ribonuclear foci, they are still capable of binding to key proteins of the alternative splicing machinery, such as MBNL1, and cause defects in RNA processing (Mykowska et al., 2011). sRNAs with as few as seven CAG repeats have been shown to induce caspase activation and cellular toxicity in a disease model of HD (Bañez-Coronel et al., 2012). It has been reported that the knockdown of Dicer expression can reduce sRNA in HD and SCA1 models, supporting the involvement of RNAi in the sRNA-induced toxicity of READs (Krol et al., 2007; Bañez-Coronel et al., 2012). Since bidirectional transcription is common with repeat expansions, the transcribed sense and antisense strands may form double-stranded RNAs (dsRNAs). Once again, dsRNA is a substrate of Dicer, thus it becomes a source of sRNAs. This mechanism was demonstrated in two *Drosophila* studies. One showed that that dsRNAs comprising CAG/CUG repeats can be a source of cellular toxicity via the Dicer pathway (Lawlor et al., 2011). The other study showed that the co-expression of CAG and CUG repeats results in enhanced RNA toxicity in a Dicer-dependent manner by generating more sRNAs, which impedes the expression of CAG-containing genes, such as *Ataxin-2* and *TATA-binding protein* (*TBP*; Yu et al., 2011). Therefore, both ssRNA hairpins and dsRNAs can be a source of harmful sRNAs that results in cellular toxicity. For hexanucleotide repeat expansions such as GGGGCC in C9-ALS/FTD, the single stranded RNA repeats can even form G-quadruplex structures (Reddy et al., 2013; Haeusler et al., 2014). G-quadruplex structures can induce a number of detrimental consequences in the cell including transcription impairment, formation of inclusions and sequestration of ribonucleoproteins to impeded alternative splicing and induce nucleolar stress (Haeusler et al., 2014; Conlon et al., 2016).

A fifth mechanism which non-coding expanded transcripts lead to cellular toxicity is by repeat-associated non-ATG (RAN) translation. Normally, protein synthesis is initiated by the start codon ATG, which leads to the translation of mRNA. Ranum and colleagues have discovered a novel mechanism which repeat expansions in READs can bypass the need of a start codon and mediate RAN translation of the repeat-containing transcripts (Zu et al., 2011). In RAN translation, the accumulated expanded repeat RNA may undergo protein translation in all three possible reading frames, resulting in the production of proteins consisting of different amino acid combinations (Zu et al., 2011). It has been shown in SCA8, FXTAS, and C9-ALS/FTD that the protein products from RAN translation are highly toxic and play an significant role in pathogenesis (Zu et al., 2011; Todd et al., 2013; Mizielinska et al., 2014; Freibaum et al., 2015; Oh et al., 2015; Tran et al., 2015; Lee et al., 2016).

A sixth mechanism of RNA toxicity involves the expanded RNAs being actively transported to neurites by transport granules, which in turn induces a neuritic branching defect in neurons (Burguete et al., 2015). This could be explained by the fact that the expanded RNA-bound transport granules cannot function normally, and the transport defects possibly interfere with the local translational machinery as well (Burguete et al., 2015).

## Modeling the RNA toxicity of repeat expansions in *Drosophila*

Recent work in *Drosophila* has begun to shed light on the emerging “RNA toxicity model.” One of the earliest *Drosophila* studies that demonstrated this model was pioneered by Rebay and colleagues in the pathogenesis of SCA8 (Mutsuddi et al., 2004). Unlike most other types of SCA, SCA8 is associated with a CTG repeat expansion within a non-coding region (Koob et al., 1999). A *Drosophila* model of SCA8 was developed by overexpressing the non-coding transcript of the human *SCA8* locus in the fly eye, which results in retinal degeneration (Mutsuddi et al., 2004). The *Drosophila* RNA binding protein genes, *staufen, muscleblind (Mbl), split ends*, and *CG3249*, were all subsequently identified as genetic modifiers of this degeneration, suggesting that RNA rather than expanded protein is the source of toxicity (Mutsuddi et al., 2004). DM1 is another non-coding READ demonstrated to be caused by RNA toxicity. DM1 is associated with a CUG repeat expansion in the 3′ UTR of the *DMPK* gene. A *Drosophila* model of DM1 was established by expressing non-coding CUG repeats in either the *Drosophila* eyes or muscles. Degeneration was observed in both cases, indicating the presence of toxic RNA gain-of-function (de Haro et al., 2006). In the case of FXTAS, a CGG repeat expansion occurs in the 5′ UTR of the *FMR1* gene. In *Drosophila*, it was reported that the expression of non-coding CGG repeats was sufficient to induce retinal degeneration in the absence of expressed proteins (Jin et al., 2003). All these studies in *Drosophila* support the notion that RNA toxicity play a significant role in the pathologies of many READs. More importantly, these studies also demonstrate the fruit fly as an excellent model for studying human READs and RNA toxicity. Currently, fly models have been established for at least 13 different READs (Table 1). Below, we will cover a number of READs with RNA toxicity and highlight the discoveries made using their corresponding *Drosophila* models. These READs were selected base on the following two criteria: (i) It has been shown that gain-of-function RNA toxicity contributes to the pathology of these diseases, and (ii) there are established *Drosophila* models for studying the RNA toxicity of these diseases.

**Table 1:** **Published transgenic ***Drosophila*** lines for modeling repeat expansion-associated diseases**.

**READ**	**Affected human gene**	**Expansion region of the human gene**	**Nucleotide repeat**	**Established *Drosophila* lines for modeling READs (under UAS control)**
HD	*Huntingtin* (*HTT*)	Coding region (Exon 1)	CAG	**With an exon1-derived HTT fragment** (Jackson et al., 1998): HTT^1−142^-Q2 HTT^1−142^-Q75 HTT^1−142^-Q120
				(Steffan et al., 2001): Htt-ex1p-Q20 Htt-ex1p-Q93 UAS-Q48 + myc/flag
				(Doumanis et al., 2009): Nhtt-18Q-EGFP Nhtt-48Q-EGFP Nhtt-98Q-EGFP Nhtt-152Q-EGFP Nhtt-48Q-EGFP^NLS^
				**With a HTT fragment of amino acids 1-336** (Kaltenbach et al., 2007): *128Q-htt*^[M64]^
				**With a HTT fragment of amino acids 1-548** (Lee et al., 2004): Htt-Q0 Htt-Q128
				**With a HTT fragment of amino acids 1-588** (Weiss et al., 2012): mRFP-Htt-Q15 mRFP-Htt-Q138 EGFP-Htt-Q15 EGFP-Htt-Q138 EGFP-Htt-Q15-mRFP EGFP-Htt-Q138-mRFP Htt^1−81^-Q96-GFP (with HTT exon 1 amino acids 1-81)
				**With full length HTT (3144 amino acids)** (Romero et al., 2008): 16Q-htt^FL^ 128Q-htt^FL^
SBMA	*Androgen receptor* (*AR*)	Coding region (N-terminus)	CAG	(Takeyama et al., 2002): AR-Q0 AR-Q52
DRPLA	*Atrophin-1* (*AT1*)	Coding region	CAG	(Nisoli et al., 2010): At-1-wt At-1-65Q At-1-65ΔC Atro-wt Atro-75QN Atro-66QC
SCA1	*Ataxin-1* (*ATXN1*)	Coding region	CAG	(Fernandez-Funez et al., 2000): Ataxin-1 Q30 Ataxin-1 Q82
SCA3 (MJD)	*Ataxin-3* (*ATXN3*)	Coding region	CAG	(Warrick et al., 1998): SCA3tr-Q27 SCA3tr-Q78
				(Warrick et al., 2005): SCA3-Q27 SCA3-Q78 SCA3-Q84 SCA3-Q27-UIM^*^ SCA3-Q80-UIM^*^ SCA3-Q27-C14A SCA3-Q88-C14A SCA3-delta
				(Li et al., 2008): SCA3tr-Q61 SCA3tr-Q78CAA/G DsRed-CAG0 DsRed-CAG100 DsRed-CAG250
SCA6	*CACNA1A*	Coding region (exon 47)	CAG	(Tsou et al., 2015): α1ACT-Q11 α1ACT-Q70
				(Tsou et al., 2016): α1ACT-Q33
SCA7	*Ataxin-7* (*ATXN7*)	Coding region	CAG	(Jackson et al., 2005): SCA7-Q90
				(Latouche et al., 2007): ATXN7T-Q10 ATXN7T-Q102
SCA8	*ATXN8OS (non-protein coding Ataxin-8 opposite strand)*	3′UTR	CTG	(Mutsuddi et al., 2004): SCA8(CTG)_9_ SCA8(CTG)_112_
SCA17	*TATA binding protein* (*TBP*)	Coding region	CAG	(Ren et al., 2011): TBP-Q34 TBP-Q54 TBP-Q80
FXTAS	*Fragile X mental retardation 1* (*FMR1*)	5′UTR	CGG	(Jin et al., 2003): (CGG)_60_-GFP (CGG)_90_-GFP
				(Todd et al., 2013): 5′UTR: (CGG)_100_-(+1)-ATG-GFP 5′ATG: ATG-(CGG)_100_-(+1)-ATG-GFP 3′UTR: ATG-GFP-STOP-(CGG)_100_-(+1) 5′Stop: STOP-(CGG)_100_-(+1)-ATG-GFP
DM1	*Dystrophia myotonica protein kinase* (*DMPK*)	3′UTR	CTG	(Houseley et al., 2005): GFP-(CTG)_11_ GFP-(CTG)_48_ GFP-(CTG)_56_ GFP-(CTG)_162_
				(de Haro et al., 2006): i(CTG)_480_
				(Garcia-Lopez et al., 2008): (CTG)_60_ i(CTG)_480_
				(Yu et al., 2011): (CTG)_19_ (CTG)_130_ (CTG)_200_ (CTG)_230_ (CTG)_250_ (CTG)_270_
				(Picchio et al., 2013): i(CTG)_240_ i(CTG)_480_ i(CTG)_600_ i(CTG)_960_ (“i” indicates “interrupted repeats”.)
DM2	*Zinc finger protein 9 (ZNF9)*	Intron 1	CCTG	(Yu et al., 2015): (CCTG)_16_ (CCTG)_200_ (CCTG)_475_ (CCTG)_525_ (CCTG)_700_ (CCTG)_720_
C9ORF72-associated ALS-FTD	*C9ORF72*	Promoter and intron 1	GGGGCC	(Xu et al., 2013): (GGGGCC)_3_ (GGGGCC)_30_
				(Mizielinska et al., 2014): (GGGGCC)_3_ Pure (GGGGCC)_36_ Pure (GGGGCC)_36_ RO (GGGGCC)_108_ RO (GGGGCC)_288_ RO (PA)_36_ (GA)_36_ (PR)_36_ (GR)_36_
				(Freibaum et al., 2015): (GGGGCC)_8_ (GGGGCC)_28_ (GGGGCC)_58_ GFP-(GP)_47_ GFP-(GA)_50_ GFP-(GR)_50_
				(Tran et al., 2015): (GGGGCC)_5R_ (flanked by human intronic and exonic sequences) (GGGGCC)_80R_ (flanked by human intronic and exonic sequences) (GGGGCC)_160R_ (flanked by human intronic and exonic sequences)

UIM

* *indicates that the highly conserved serine resides in the ubiquitin interaction motifs are mutated to alanine to disrupt ubiquitin binding*.

### *Drosophila* models of polyglutamine diseases

Polyglutamine diseases are caused by the expansion of unstable CAG repeats in the protein-coding region of the respective disease gene. The CAG repeats are translated into a string of glutamine in normal functioning proteins. Patients of polyQ diseases often develop progressive neurodegeneration and motor impairment (Rub et al., 2013). PolyQ diseases include HD, spinobulbar muscular atrophy (SBMA; a.k.a. Kennedy disease), dentatorubral-pallidoluysian atrophy (DRPLA), spinocerebellar ataxia (SCA) types 1, 2, 3 (a.k.a. Machado-Joseph disease or MJD), 6, 7, and 17 (La Spada et al., 1991; MacDonald et al., 1993; Orr et al., 1993; Kawaguchi et al., 1994; Imbert et al., 1996; Pulst et al., 1996; Sanpei et al., 1996; David et al., 1997; Zhuchenko et al., 1997; Nakamura et al., 2001; Warrick et al., 2005). HD is caused by an abnormal expansion of CAG repeats in exon 1 of the *Huntingtin* (*HTT*) gene (MacDonald et al., 1993). For SBMA, the CAG repeat expansion lies in the N-terminus of the coding region of the androgen receptor gene (La Spada et al., 1991; Zhou et al., 1994). For DRPLA, the unstable CAG expansion occurs in the middle of the Atrophin-1 gene (Koide et al., 1994). As for SCA1, 2, 3, 6, 7, and 17, the CAG expansion are located in the coding regions of *Ataxin-1, Ataxin-2, Ataxin-3, CACNA1A, Ataxin-7*, and *TATA-binding protein* (*TBP*), respectively (Nakamura et al., 2001; Rub et al., 2013). Despite the fact that the CAG repeat expansions in these polyQ diseases affect different genes, there are several shared features among the majority of polyQ diseases. First, a common hallmark is the abnormal aggregation of nuclear inclusions containing the mutant proteins. These inclusions that contain abnormal aggregated proteins were once regarded as a potential cause of cellular toxicity. However, a number of studies have demonstrated that the formation of inclusions could actually be a cellular protective response to mitigate the toxicity (Stefanis et al., 2001; Petrucelli et al., 2002; Arrasate et al., 2004). Until today, the general belief is that the formation of aggregates is a dynamic process. Smaller oligomeric inclusions are likely to toxic, whereas the large aggregates might actually be protective (Ross and Poirier, 2005). Further investigations will be required to elucidate their true nature. Second, the CAG repeat length is inversely proportional to the age of disease onset. Longer CAG lengths cause earlier onset of polyQ diseases (Zoghbi and Orr, 2000; Ross and Poirier, 2005). This is because once the polyQ repeat length exceeds a pathogenic threshold (typically ~35), the mutant proteins are likely to mis-fold into abnormal conformations that can resist normal cellular degradation machineries such as the ubiquitin-proteasome system (Sisodia, 1998; Chan et al., 2000; Ross and Poirier, 2005).

PolyQ diseases were among the first neurodegenerative diseases modeled in *Drosophila*. The earliest *Drosophila* models include HD and SCA3. Zipursky and colleagues fused a truncated form of wild-type human HTT with a polyQ tract of 2, 75, or 120 repeats (HTT^1−142^-Q2, HTT^1−142^-Q75 and HTT^1−142^-Q120; Jackson et al., 1998), whereas Bonini and colleagues generated truncated forms of human Ataxin-3 with a polyQ tract of 27 or 78 repeats (SCA3tr-Q27 and SCA3tr-Q78) (Warrick et al., 1998). When expressed in the fly eye, both studies found that only the constructs with pathogenic lengths (HTT^1−142^-Q75, HTT^1−142^-Q120, and SCA3tr-Q78) induced nuclear inclusions and degeneration, recapitulating the pathogenic features of polyQ diseases. These studies, for the first time, demonstrated that *D. melanogaster* can be a powerful model to study human neurodegenerative diseases. Subsequently, more *Drosophila* models of HD and SCA3 (Steffan et al., 2001; Lee et al., 2004; Warrick et al., 2005; Kaltenbach et al., 2007; Li et al., 2008; Romero et al., 2008; Doumanis et al., 2009; Weiss et al., 2012), as well as other polyQ diseases, including SCA1 (Fernandez-Funez et al., 2000), SCA6 (Tsou et al., 2015, 2016), SCA7 (Jackson et al., 2005; Latouche et al., 2007), SCA17 (Ren et al., 2011), SBMA (Takeyama et al., 2002; Pandey et al., 2007; Nedelsky et al., 2010), and DRPLA (Nisoli et al., 2010; Napoletano et al., 2011) were developed. All these studies demonstrate that polyQ diseases, in most cases, can yield parallel neural degenerative effects in the fruit fly.

Besides protein toxicity, there is now substantial evidence for RNA toxicity in polyQ diseases as well. One of the most compelling studies was performed by Bonini and colleagues in the studying of SCA3 in *Drosophila* (Li et al., 2008), which they showed that the expression of both translated and untranslated CAG repeats caused neural degeneration, whereas the expression of CAA-interrupted CAG repeats resulted in significantly less pronounced neurodegenerative effects (Li et al., 2008). Similar to CAG repeats, CAA repeats also encode for glutamine. Yet CAA repeats are unable to form a hairpin structure (Sobczak et al., 2010). As the hairpin structure contributes to CAG repeat-containing sRNA generation, it is one of the major mechanisms of RNA toxicity (Krol et al., 2007; Bañez-Coronel et al., 2012). Therefore, the above findings suggest that RNA toxicity contributes significantly to neurodegeneration in SCA3.

Apart from hairpin structures, it is also known that CAG repeats in polyQ diseases, similar to CUG repeats in DM1, can form ribonuclear foci that colocalize with RNA-binding proteins such as muscleblind-like 1 (MBNL1), and cause misregulation in alternative splicing (Ho et al., 2004, 2005; Yuan et al., 2007; Hsu et al., 2011; Mykowska et al., 2011; Wang et al., 2011). In fact, Krzyzosiak and colleagues demonstrated that expanded CAG repeats present in HTT and ATXN3 transcripts colocalize with MBNL1 in ribonuclear foci, further confirming the accumulation of mutant RNA in the nucleus (Mykowska et al., 2011). Interestingly, although both CAG and CUG repeats form foci, sequester MBNL1 and affect alternative splicing, the splicing of some pre-mRNAs are only affected by CUG repeats but not CAG repeats (Ho et al., 2005). Hence, despite the overlapping mechanisms of involvement of MBNL1, there are still differences in how splicing is disrupted by CAG repeats in polyQ diseases and CUG repeats in DM1.

In addition to the sequestering of RNA-binding proteins, RNA toxicity in polyQ diseases may also arise from the perturbation of RNA export mechanisms and the induction of nucleolar stress (Tsoi et al., 2011, 2012). In the *Drosophila* model of SCA3, it has been demonstrated that U2AF65, which is involved in the nuclear export of mRNA, can bind directly to CAG repeats and form a complex with the nuclear export receptor NXF1 (Tsoi et al., 2011), and the downregulation of U2AF65 correlates with the nuclear accumulation of expanded CAG RNA (Tsoi et al., 2011). Apart from U2AF65, CAG repeat expansion can also interact directly with NCL, and induce apoptosis by the activation of the nucleolar stress pathway (Tsoi et al., 2012). NCL is an essential protein involved in RNA polymerase I-mediated rRNA transcription in the nucleolus. The binding of expanded CAG repeats perturbs ribosome biogenesis, which in turn increases the levels of mitochondrial p53, and causes cytochrome c release and caspase activation (Tsoi et al., 2012).

Utilizing the established fly models of SCA3, much advances have been made in the search of genetic modifiers of polyQ toxicity and CAG-repeat RNA toxicity. Using the fly model of SCA3, HSP70 was found to colocalize with polyQ inclusion bodies, and was identified as a strong suppressor of polyQ toxicity (Warrick et al., 1999; Chan et al., 2000). A genome-wide genetic screen was also performed, and identified a number of other chaperones and ubiquitin pathway-related molecules as genetic modifiers of polyQ toxicity (Bilen and Bonini, 2007). As for RNA toxicity in SCA3, a microarray analysis was performed to search for genes which expression levels are affected by CAG repeats (Shieh and Bonini, 2011). Several iron ion binding and nucleotide binding molecules were found to be affected. Interestingly, HSP70, co-chaperone Tpr2, the transcriptional regulator Dpld, and the RNA-binding protein Orb2 were able to modify both polyQ and RNA toxicity, suggesting that there are overlapping mechanisms of RNA and protein based toxicity (Shieh and Bonini, 2011).

All the above studies demonstrated that both protein and RNA toxicity contribute to neurodegeneration in polyQ diseases, and both types of toxicity can involve a number of different underlying molecular mechanisms which can be studied using *Drosophila* models.

### *Drosophila* models of fragile X syndrome and fragile X-associated tremor/ataxia syndrome

Fragile X syndrome (FXS) is the most common cause of inherited intellectual disability, often associated with autism spectrum disorders (Bhakar et al., 2012; Santoro et al., 2012). The disease was first described as mental retardation segregated in X-linked fashion (Martin and Bell, 1943). The chromosomal variation was later mapped to Xq27.3 and was dubbed “the fragile X chromosome” (Harrison et al., 1983). In FXS, a trinucleotide CGG expansion in the 5′ UTR of the *FMR1* gene results in transcriptional silencing and loss of the corresponding protein, Fragile X Mental Retardation Protein (FMRP; Fu et al., 1991; Kremer et al., 1991). Normal alleles contain 6–55 repeats, premutation alleles contain 55–200, and the disease alleles contain over 200 repeats. FMRP is an RNA-binding protein which negatively regulates the translation of mRNAs, especially at synapses in neurons. Loss of FMRP impairs synaptic plasticity, which is believed to be the molecular basis for the intellectual disability in FXS patients (Bassell and Warren, 2008).

The *Drosophila* genome contains a highly-conserved *FMR1* gene, *dfmr1* (Wan et al., 2000). The first *Drosophila* FXS model was generated by imprecise excision of a *P*-element located upstream of the *dfmr1* locus to produce a null mutant (Zhang et al., 2001). Subsequently, a wide array of *dfmr1* alleles and transgenes have been created to facilitate FXS research in this classic genetic model (Dockendorff et al., 2002; Morales et al., 2002; Lee et al., 2003). Overexpression of either the human FMRP (*FMR1* gene) or the native *Drosophila* dFMRP (*dfmr1* gene) fully rescues all detectable disease-like phenotypes in the *Drosophila* FXS model (Coffee et al., 2010), confirming the evolutionary conserved functions of *FMR1* in the nervous system.

In 2003, Warren and colleagues expressed 90 CGG repeats with a reporter in the *Drosophila* compound eye and demonstrated retinal degeneration in the absence of expressed proteins (Jin et al., 2003). These findings suggest that, apart from loss-of-function of wild-type FMRP, there exists a toxic RNA gain-of-function component associated with the repeat expansion that occurs at the 5′ UTR of the *FMR1* gene. This RNA toxicity causes a late-onset neural degeneration phenotype, which is now identified as Fragile X-associated tremor/ataxia syndrome (FXTAS). While FXS is mostly caused by the loss-of-function of wild-type FMRP, which results in neurodevelopmental and behavioral disorders characterized by mental retardation, autism, anxiety and mood instability, FXTAS is mostly caused by the CGG repeat expansion that produces gain-of-function RNA toxicity in the CNS, which results in a late-onset neurodegenerative disorder, characterized by progressive intention tremor, gait ataxia, parkinsonism, and cognitive decline (Jacquemont et al., 2007; Tassone and Hagerman, 2012). FXTAS is found mostly in male premutation carriers after the age of 50 (Tassone and Hagerman, 2012). Although at a much lower rate, female carriers may also develop FXTAS. The neuropathological hallmark of FXTAS is the ubiquitin-positive intranuclear inclusion, present in both neurons and astrocytes throughout the brain (Greco et al., 2002). Warren and colleagues found that in their (CGG)_90_ fly model of FXTAS, the inclusion bodies in the degenerating neurons contain HSP70 (Jin et al., 2003). Furthermore, when they overexpress HSP70, the CGG repeat-induced degeneration was suppressed (Jin et al., 2003), which is similar to the case of SCA3 where HSP70 also presents suppressor activity of CAG repeat-induced toxicity (Warrick et al., 1999; Chan et al., 2000). Two subsequent studies have utilized this *Drosophila* model of FXTAS and identified a number of tropomyosin and RNA-binding proteins as genetic modifiers of the degeneration phenotype (Jin et al., 2007; Sofola et al., 2007). These findings strongly suggest that the RNA processing machinery play an important role in the pathogenesis of FXTAS. Interestingly, both studies have found that one of these RNA-binding proteins that interacts with the CGG repeats to be CUG-binding protein 1 (CUGBP1), which is a protein also involved in the pathogenesis of another READ, DM1. Indeed, CGG repeats have been shown to form RNA hairpin structures *in vitro*, similar to CAG repeats in SCA3 and CUG repeats in DM1 (Sobczak et al., 2003, 2010). Thus, it is possible that these RNA-binding proteins bind to the CGG repeats in an effort to disassemble these potentially toxic hairpins, but in the process neglected their other cellular functions.

Recent studies in neurodegenerative and neuromuscular diseases have advanced toward the investigation of cellular transcriptional and translational profiles. Nelson and colleagues discovered that mice Purkinje neurons ectopically expressing 90 CGG repeats exhibit a dramatic change in their translational profile even prior to the onset of CGG repeat-induced phenotypes (Galloway et al., 2014). Interestingly, the *Tardbp* gene, which encodes the TDP-43 protein was found to be reduced in Purkinje neurons (Galloway et al., 2014). TDP-43 is a RNA-binding protein commonly found in the inclusion bodies in ALS (Xu and Yang, 2014). In the fly model of FXTAS, overexpression of TDP-43 suppresses neurodegeneration in the fly eye, whereas knockdown of *Drosophila* TDP-43 results in enhancement of the degeneration phenotype (Galloway et al., 2014). Todd and colleagues also discovered that overexpression of TDP-43 was capable of suppressing CGG repeat toxicity (He et al., 2014). Interestingly, they found that the CGG repeat RNA levels and RAN translation levels are both not affected by TDP-43. Instead, they found TDP-43 alters the composition, behavior and distribution of multimeric RNA-binding protein complexes, preventing the sequestration of hnRNP A2/B1, and rescuing the alternative splicing machinery (He et al., 2014). They also tested an ALS mutant form of TDP-43, which can still bind to hnRNP A2/B1, and yet it failed to rescue the CGG repeat toxicity, suggesting that wild type TDP-43 functions are necessary for this suppression of CGG repeat toxicity (He et al., 2014).

In the study above by Nelson and colleagues, the researchers discovered ~500 transcripts that are differentially associated with ribosomes in the CGG repeats-expressing Purkinje neurons (Galloway et al., 2014). Theoretically, if ribosomes are recruited to the repeat expansions, translation of the repeats would be facilitated. Indeed, RAN translation has also been demonstrated in FXTAS models. Paulson and colleagues have demonstrated that CGG repeats lead to RAN translation of a cryptic polyglycine-containing protein, FMRpolyG, which is present in inclusion bodies in FXTAS fly models, mouse models and FXTAS patient brains (Todd et al., 2013). The researchers constructed a *Drosophila* line expressing CGG repeats preceded by a stop codon just 5′ to the repeats. Production of FMRpolyG is inhibited by this stop codon, but the CGG repeat-containing RNA remained. This line gave rise to a much milder phenotype comparing with CGG repeats without the stop codon, indicating RAN translated FMRpolyG contribute to toxicity (Todd et al., 2013). A follow-up *Drosophila* study revealed that FMRpolyG impairs the ubiquitin protease system (Oh et al., 2015). Inhibition of RAN translation can reduce inclusion formation and attenuate the impairment of the ubiquitin protease system (Oh et al., 2015).

Taken together, these studies suggest that FXTAS pathogenesis may be mechanistically related to the polyQ diseases, myotonic dystrophy, and ALS. Thus, different READs that arise from different causes may converge and share some common molecular mechanisms in their pathogenesis.

### *Drosophila* models of myotonic dystrophy type 1 and type 2

Myotonic dystrophy is the most common form of muscular dystrophy affecting about 1 in 8,000 adults (Harper, 2001). It is a multisystemic, autosomal dominant neuromuscular disorder, characterized by progressive myotonia (inability of muscles to relax after contraction), muscle degeneration, iridescent cataracts, cardiac arrhythmias, testicular atrophy, and symptoms of neuropathology (Harper, 2001). DM1 is caused by a CTG repeat expansion in the 3′ UTR of the *DMPK* gene, which encodes a serine-threonine protein kinase (Brook et al., 1992). Normal individuals typically have fewer than 37 CTG repeats, whereas DM1 patients can range from 50 to 4,000 repeats (Thornton et al., 1994). The mutant transcripts containing CUG repeats fold into RNA hairpins that are retained in the nucleus (Davis et al., 1997; Koch and Leffert, 1998), whereas the levels of DMPK protein are correspondingly reduced (Fu et al., 1993). *Dmpk* knockout mice have reduced force generation in skeletal muscles (Reddy et al., 1996) and abnormal cardiac conduction (Berul et al., 1999), which suggests that the loss of DMPK function may contribute to at least the muscle weakness and cardiac defects in DM1. However, *Dmpk* knockout mice do not display myotonia and muscle degeneration (Reddy et al., 1996), which are some of the hallmark features of DM1. In contrast, transgenic mice expressing expanded CTG repeats display myotonia, degeneration, and several other muscular pathological abnormalities (Mankodi et al., 2000). These results demonstrated that the expanded CTG repeats by themselves could account for at least some of the major pathological features of DM1 independent of the *DMPK* locus. It was eventually demonstrated that the CUG-repeat-containing transcripts retained in the nucleus aggregate into ribonuclear foci (Taneja et al., 1995; Mankodi et al., 2000). These foci binds to RNA-binding proteins like MBNL1 and CUGBP1 (Miller et al., 2000; Kanadia et al., 2003), which in turn compromises the RNA splicing machinery (Philips et al., 1998; Ho et al., 2004) and even disrupts the regulation of transcription (Ebralidze et al., 2004).

Monckton and colleagues developed a *Drosophila* model of DM1 by fusing 162 non-coding CTG repeats to a reporter gene and overexpressing the repeats in fly muscles (Houseley et al., 2005). Although 162 CUG repeats results in the formation of ribonuclear foci, it did not result in detectable cellular toxicity. Botas and colleagues further examined the effect of non-coding CUG repeats by increasing the repeat number to 480 (interrupted repeats). With this amount of repeats, vacuolization and degeneration were then detected in adult fly muscles (de Haro et al., 2006). Expression of 480 CUG repeats in the adult eye also results in disorganization of ommatidia and reduction of the eye size, suggesting gain-of-function RNA toxicity (de Haro et al., 2006; Garcia-Lopez et al., 2008). Similar to mammals, the molecular mechanisms of this CUG-induced toxicity involve the sequestration of *Drosophila* Mbl and CUGBP1 by the CUG repeat-containing ribonuclear foci, resulting in defective alternative splicing (de Haro et al., 2006; Garcia-Lopez et al., 2008). However, in contrast to CUGBP1, which has been shown to interact with CGG repeats and is involved in FXTAS, no evidence for the genetic interaction of muscleblind family proteins and CGG repeats had been found in *Drosophila* (Sofola et al., 2007). Thus, although the pathogenesis of FXTAS and myotonic dystrophy may share some commonalities, there are certainly distinct mechanisms for the two READs as well. Utilizing this fly model of DM1, Botas and colleagues identified SMAUG, a myosin-binding protein, as suppressor of CUG repeat-induced toxicity (de Haro et al., 2013). SMAUG interacts genetically and physically with CUGBP1, and its overexpression restores the translational activity of CUGBP1 in this DM1 model (de Haro et al., 2013).

Refinements of the DM1 fly models were made in some recent studies, with new discoveries being yield. Jagla and colleagues constructed fly lines with 240, 480, 600, and 960 interrupted CTG repeats, and established a novel *Drosophila* larval model of DM1 (Picchio et al., 2013). Using this model, they constructed transcription profiles of DM1, and uncovers a number of splice-independent deregulated genes (Picchio et al., 2013). Bonini and colleagues cloned 19, 130, 200, 230, 250, and 270 uninterrupted, pure CTG repeats into the 3′ UTR of the *DsRed* gene to better recapitulate the pathological condition of the disease (Yu et al., 2011). Repeat length-dependent toxicity was detected when the repeat length reaches 200 or more. Interestingly, the researchers discovered that there was a synergistic effect on the enhancement of toxicity when CAG repeats are co-expressed together with CUG repeats due to the biogenesis of sRNAs (Yu et al., 2011). This enhancement was Dicer-dependent, indicating the involvement of the RNAi machinery (Yu et al., 2011).

DM2 is caused by a tetranucleotide CCTG repeat expansion in the first intron of the *zinc finger protein 9* (*ZNF9*) gene, which encodes a RNA-binding protein (Liquori et al., 2001). Affected individuals can have 75 to 11,000 CCTG repeats, with a mean of ~5,000 (Liquori et al., 2001). DM2, much like DM1, involves the aggregation of expanded mutant transcripts into ribonuclear foci, which interfere with key molecules of the splicing machinery, such as MBNL1 and CUGBP1 (Liquori et al., 2001). Although DM1 and DM2 share much similarities in their pathologies, DM2 has less-pronounced neurological involvement and cannot be congenital (Minnerop et al., 2011). To develop a fly model of DM2, Bonini and colleagues generated constructs with 16, 200, 475, 525, 700, and 720 non-coding CCTG repeats to investigate length-dependent RNA toxicity (Yu et al., 2015). Expression of 200 repeats was sufficient to induce the formation of ribonuclear foci, but impairment of alternative splicing and retinal degeneration were only observed when the repeat length was 475 or above. In addition, the researchers demonstrate that the expression of two different isoforms of MBNL1 (with or without the linker region between zinc finger domain 2 and 3) can lead to cleavage of non-coding CUG or CCUG RNA repeats, and mildly rescue CCUG-induced degeneration in the fly eye (Yu et al., 2015).

### *Drosophila* models of C9ORF72-associated amyotrophic lateral sclerosis/frontotemporal dementia

Amyotrophic lateral sclerosis (ALS), also known as Lou Gehrig's disease, is an adult-onset neurodegenerative disease characterized by the progressive loss of motor neurons in the cortex, brainstem, and spinal cord. Symptoms include the loss of movement control, difficulty in speaking, swallowing, and eventually breathing, which leads to fatality (Kurtzke, 1982). ALS affects about 2 per 100,000 individuals in Europe and the United States (Kiernan et al., 2011). Most cases of ALS are deemed sporadic, but 5–10% cases are inherited (Hand and Rouleau, 2002). Mutations from more than 20 different genes have been linked to familial ALS (Chen et al., 2013; Ajroud-Driss and Siddique, 2015). Despite some of these identified genes play a role in RNA metabolism and axonal trafficking (Strong, 2010; Ikenaka et al., 2012; Millecamps and Julien, 2013; Droppelmann et al., 2014), most of them apparently do not function in the same molecular pathway, complicating the underlying mechanisms of the disease (Chen et al., 2013; Ajroud-Driss and Siddique, 2015).

In 2011, the expansion of a GGGGCC hexanucleotide repeat in the non-coding region of *C9ORF72* was found to be associated with ALS combined with frontotemporal dementia (DeJesus-Hernandez et al., 2011; Renton et al., 2011). Frontotemporal dementia is a group of disorders caused by the progressive loss of neurons predominantly at the frontal and/or temporal lobes of the brain resulting in cognitive and behavioral impairment, and is one of the most common causes of dementia second only to Alzheimer's disease (Mercy et al., 2008; Perry and Miller, 2013; Baizabal-Carvallo and Jankovic, 2016). Remarkably, over 6% of sporadic ALS/FTD cases and over 37% familial ALS/FTD cases are caused by the expansion of the GGGGCC hexanucleotide repeat in the first intron of the *C9ORF72* gene (Majounie et al., 2012).

Jin and colleagues pioneered the first *Drosophila* model of C9-ALS/FTD by expressing 30 GGGGCC repeats [(GGGGCC)_30_] with a reporter in the compound eye, which resulted in severe retinal degeneration (Xu et al., 2013). When (GGGGCC)_30_ was expressed in motor neurons, it resulted in defective locomotor activity; whereas when it was expressed in the central nervous system using a pan-neuronal driver, it resulted in lethality (Xu et al., 2013). One important question to be answered was how GGGGCC repeats actually cause the disease. The pathogenesis could be due to: (1) loss-of-function of wild-type C9ORF72 protein, (2) gain-of-function of toxic RNA consisting of the GGGGCC repeats, and/or (3) gain-of-function of toxic dipeptide repeat (DPR) proteins generated by RAN translation. Although the mild phenotypes in a homozygous *C9ORF72* mutation case and the lack of *C9ORF72* coding mutations suggest that wild-type protein loss-of-function is unlikely the primary cause of C9-ALS/FTD (Fratta et al., 2013; Harms et al., 2013), loss-of-function of the wild type protein is still a possible mechanism that contribute to the disease pathogenesis. Since *Drosophila* does not have an ortholog of C9ORF72, the fly may not be a suitable model organism for investigating the haploinsufficiency aspect of C9ORF72. The fly, however, proved a valuable tool in determining the gain-of-function toxicity of GGGGCC repeats in C9-ALS/FTD. In the attempt to differentiate between repeat RNA and DPR protein toxicity, Isaacs and colleagues generated stop codon-interrupted RNA-only (RO) repeats of GGGGCC and compared it with pure GGGGCC repeats which can be RAN-translated into DPR proteins in *Drosophila* (Mizielinska et al., 2014). Isaacs and colleagues found that, the expression of RO (GGGGCC)_36_ or pure (GGGGCC)_36_ results in the same amount of ribonuclear foci formation. However, the expression of all RO constructs, including RO (GGGGCC)_36_, RO (GGGGCC)_108_, and even RO (GGGGCC)_288_, had no detectable effect on the fly eye. In contrast, both pure (GGGGCC)_36_ and pure (GGGGCC)_103_ caused degeneration. These findings suggest that, although both RNA and DPR proteins may contribute to toxicity, degeneration was primarily attributable to DPR proteins (Mizielinska et al., 2014). To further investigate whether DPR protein expression alone was sufficient to cause degeneration, they generated four “protein-only” constructs by using alternative codons. When expressed in the fly eye, only glycine-arginine (GR)_36_ and proline-arginine (PR)_36_ constructs resulted in degeneration, whereas glycine-alanine (GA)_36_ and proline-alanine (PA)_36_ had no detectable effect. Even at the repeat length of 100, same results were obtained for the above DPR proteins, indicating that high arginine-containing DPR protein is the major cause of neurodegeneration in C9-ALS/FTD (Mizielinska et al., 2014).

To determine the molecular mechanism that underlies DPR proteins-induced degeneration, Taylor, Gao and colleagues generated reporter-tagged (GGGGCC)_58_ flies, and performed a screen to look for genetic modifiers of GGGGCC-induced eye degeneration (Freibaum et al., 2015). Eighteen modifier genes were identified with the majority of them being transcription/export (TREX) complex proteins and nuclear pore complex proteins, involving particularly nuclear export of RNA. Lastly, Taylor, Gao and colleagues predicted that (GGGGCC)_58_ can produce poly(GA), poly(GR), or poly(GP) DPR proteins by RAN translation. Consistent with an aforementioned study, they found that the arginine-containing poly(GR) to be highly toxic (Freibaum et al., 2015). In summary, the researchers demonstrated that GGGGCC repeat expansion compromises nucleocytoplasmic transport, facilitating nuclear retention of toxic RNAs. Whereas the build-up of toxic poly(GR) DPR proteins by RAN translation is a major cause of GGGGCC-induced neurodegeneration. Rothstein and colleagues also conducted a screen to search for interactors for GGGGCC RNA, and uncovered RanGAP as a potent suppressor of neurodegeneration (Zhang et al., 2015). RanGAP is a key regulator of nucleocytoplasmic transport, supporting that the nucleocytoplasmic transport mechanisms are involved in the neurodegeneration in C9-ALS/FTD (Zhang et al., 2015). In the same year, another screen performed in yeast by Gitler and colleagues identified effectors of Ran-mediated nucleocytoplasmic transport and karyopherins as modifiers of GGGGCC-induced DPR protein toxicity (Jovicic et al., 2015). A recent study by Van Den Bosch and colleagues also reported the results of a *Drosophila* genetic screen, which identified importins, exportins, Ran-GTP cycle regulators, nuclear pore components and arginine methylases as potent modifiers of DPR protein toxicity (Boeynaems et al., 2016). Taking a different approach, Taylor and colleagues analyzed the interactome of all DPR proteins in human cells, and found that arginine-containing DPR proteins (GR and PR) interact with RNA-binding proteins with low complexity sequence domains (LCDs), such as TDP-43, FUS, hnRNPA1, hnRNPA2B1, Matrin-3, and Ataxin-2 (Lee et al., 2016). Using the *Drosophila* eye, they confirmed that these proteins are genetic modifiers of DPR protein-induced toxicity. Remarkably, most of these genetic modifiers encode components of membrane-less organelles, including the nucleoli, the nuclear pore complexes and stress granules. The researchers showed that GR and PR bind to the LCD-containing RNA-binding proteins, alter their biophysical properties, and lead to impairment of their assembly, dynamics, and functions (Lee et al., 2016).

A consensus from all these studies was that the DPR proteins that cause toxicity in C9-ALS/FTD contain arginine. Arginine is a unique amino acid that has as many as five potential hydrogen-bond donors that are readily available to react with biological hydrogen-bond acceptors. In DNA-protein complexes, arginine residues are the most frequent hydrogen bond donors to backbone phosphate groups (Luscombe et al., 2001). Networks of hydrogen bonds can be formed by arginine residues with phosphate groups of RNA loops as well (Calnan et al., 1991). Whereas among the interactions between amino acids, the arginine-aspartate double hydrogen-bond interaction is also an extremely stable structure (Mitchell et al., 1992). Each methylation of an arginine residue removes a potential hydrogen-bond donor, and changes the shape of the amino acid. Thus, methylation can reduce the chance of arginine bonding to its interactors, which in turn increases the chance of an arginine-containing DPR protein to be degraded. This is supported by the finding that arginine methylases are potent modifiers of DPR protein toxicity (Boeynaems et al., 2016).

Arginine-containing DPR proteins have been proven toxic in a number of studies by various groups. But the question remained whether the nuclear GGGGCC repeat-containing RNA induces toxicity. The study above by Isaacs and colleagues did not detect any significant degeneration when as many as 288 repeats (RO) were expressed in the fly eye (Mizielinska et al., 2014). Gao and colleagues further explored this important question, and tested the effects of a *Drosophila* model expressing a *C9ORF72* minigene with 160 GGGGCC repeats (160R; Tran et al., 2015). The 160R in this model is flanked by human intronic and exonic sequences of *C9ORF72*. Intriguingly, even though the spliced intronic 160R formed abundant nuclear GGGGCC sense RNA foci, they gave rise to no detectable neurodevelopmental defects. Cells were also normal in their rRNA biogenesis and mRNA processing (Tran et al., 2015). When 160R flies were reared at 29°C instead of the normal 25°C, a modest level of toxicity was detected. But since the higher temperature induced 4-folds more DPR proteins, while the number of RNA foci did not change, it is likely that this toxicity was due to the increased DPR proteins instead of the RNA foci (Tran et al., 2015).

Summarizing the above studies, the GGGGCC repeat expansion in C9-ALS/FTD share much commonalities with other READs such as the formation of foci, sequestration of RNA-binding proteins, and induction of nucleolar stress. Yet, unlike most other READs described here, the RNA transcripts containing the GGGGCC repeat expansions and the foci in C9-ALS/FTD do not appear to be the main source of toxicity. Rather, it is the DPR proteins produced by RAN translation (which can still arguably be called a type of RNA toxicity) that are the major determinants of toxicity and degeneration in this disease.

Nevertheless, just when everyone thought GGGGCC repeat-containing RNA does not give rise to any kind of toxicity, Bonini and colleagues discovered a new form of GGGGCC RNA toxicity that may be independent of DPR proteins. In cultured mice spinal cord neurons, GGGGCC and CAG repeat RNAs were found to localize to transport granules that travel to distal neuritic segments (Burguete et al., 2015). Expression of the GGGGCC repeat-containing RNA gave rise to neuritic branching defects. Using a *Drosophila* model, the researchers found that this branching defects can be enhanced by the upregulation or suppressed by the downregulation of *dfmr1* or *orb2* (Burguete et al., 2015). The researchers speculated that the presence of GGGGCC repeat RNA in neurites might result in local RAN translation of toxic DPR proteins. However, they were unable to detect RAN peptides in their study (Burguete et al., 2015). These findings demonstrate that GGGGCC expanded repeat RNAs can cause transport granule dysfunction, which could be a novel RNA toxicity mechanism independent of DPR proteins, contributing to the neuronal defects in C9-ALS/FTD.

It is important to note that, hexanucleotide repeats such as GGGGCC, are capable of forming distinct structures called DNA and RNA G-quadruplexes (Reddy et al., 2013; Haeusler et al., 2014). These structures facilitate DNA/RNA hybrids, impedes transcription and binds to ribonucleoproteins such as NCL, inducing nucleolar stress (Haeusler et al., 2014). They also form inclusions and sequester hnRNP H, which impedes alternative splicing (Conlon et al., 2016). The real impacts of these G-quadruplex structures on the pathogenesis of C9-ALS/FTD are only just beginning to be elucidated.

## Therapeutic development to combat RNA toxicity in reads

Much progress has been made in the past decade regarding potential therapeutic strategies against the RNA toxicity in READs. The current three main streams of therapeutics are oligonucleotide-based therapeutics, peptoid-based therapeutics, and small molecule-based therapeutics. In oligonucleotide-based therapeutics, antisense-oligos (ASOs) have been demonstrated to disrupt foci formation and reduce RNA toxicity in both DM1 and C9-ALS/FTD (Mulders et al., 2009; Wheeler et al., 2012; Donnelly et al., 2013; Lagier-Tourenne et al., 2013; Sareen et al., 2013; Riboldi et al., 2014; Wojtkowiak-Szlachcic et al., 2015). Peptoid-based therapeutics have shown some promising results in mitigating RNA toxicity in DM1 models by reducing the interaction between CUG repeats and MBNL1 and decreasing foci formation (Pushechnikov et al., 2009; García-López et al., 2011; Rzuczek et al., 2013). It has also been reported that a 13-amino-acid peptide can bind to CAG repeats and suppress nucleolar stress and neurotoxicity in a *Drosophila* model of SCA3 (Zhang et al., 2016). Last but not least, designs in small molecules designs have achieved much successes in combating DM1 and C9-ALS/FTD as well. Non-steroidal anti-inflammatory drugs like ketoprofen, histamine receptor inhibitors like orphenadrine and sodium/calcium metabolism modifiers like clenbuterol and spironolactone have been shown in *Drosophila* models of DM1 to mitigate the effects of CUG toxicity (Garcia-Lopez et al., 2008). Pentamidine and neomycin B have been shown to disrupt MBNL1 binding to CUG repeats (Warf et al., 2009). A number of small molecules, including bisamidinium inhibitors, can target toxic RNA-protein interactions and alleviate the sequestration of MBNL1 by repeat expansions (Gareiss et al., 2008; Kumar et al., 2012; Parkesh et al., 2012; Wong et al., 2014; Luu et al., 2016). Porphyrins that distorts RNA G-quadruplex structures can ablate the GGGGCC repeat expansion's interaction with hnRNPA1 or ASF/SF2 (Zamiri et al., 2014). There are small molecules that can even reduce DPR protein production in a model of C9-ALS/FTD (Su et al., 2014). Histone deacetylase (HDAC) inhibitors like sodium butyrate and suberoylanilide hydroxamic acid had been shown to suppress degeneration in a *Drosophila* model of HD (Steffan et al., 2001). Although, this suppression may involve protein toxicity instead of RNA toxicity. Furthermore, in a *Drosophila* model of SBMA and a *Drosophila* model of FXTAS, overexpression of the HDACs themselves actually suppressed the degeneration phenotypes (Pandey et al., 2007; Todd et al., 2010). Thus, the effects of HDACs inhibitors could be different for different types of READs.

The newest trend in the designing of small molecules is driving toward compounds with multiple modes of actions in combating the pathogenesis of the disease. Zimmerman and colleagues have reported a type of small molecule can target DM1 pathology by acting as transcription inhibitors, by inhibiting aberrant protein binding to toxic RNA, and by acting as RNase mimics to degrade the toxic RNA (Nguyen et al., 2015). Gitler and colleagues discovered that the transcription elongation factor Spt4 selectively decreased both sense and antisense GGGGCC expanded transcripts, and partially rescued retinal degeneration in the fly eye model of C9-ALS/FTD (Kramer et al., 2016). Knockdown of *SUPT4H1*, the human ortholog of *Spt4*, decreased sense and antisense RNA foci and DPR protein production in C9-ALS/FTD patient-derived cells (Kramer et al., 2016). The study demonstrated Spt4 as a new potential therapeutic target since the disruption of Spt4 can ameliorate degeneration induced by different modes of toxicity sources. Animal models such as the fly models described in this review are valuable tools in the testing of novel compounds and therapeutic targets before clinical trials. They will also continue to serve as important platforms for the discoveries of unknown mechanisms of diseases that may facilitate the development of new therapeutic approaches.

## Concluding remarks

In this review, we highlighted *Drosophila* as an excellent model organism for the studying of READs. The studies mentioned above have revealed comprehensive information regarding the mechanisms of neurodegeneration in READs, demonstrating the elegant use of the fly models in pioneering discoveries of RNA toxicity. Despite the fact that protein toxicity is a major factor that contributes to the pathogenesis of many READs, we are now certain that RNA toxicity also plays a major role in inducing neural and muscle degeneration via a number of possible ways, including but not limited to the impairment of alternative splicing, the promotion of bidirectional transcription, the induction of nucleolar stress and disruption of nucleocytoplasmic transport, the generation of sRNA from hairpins and dsRNA, and RAN translation of repeat-containing peptides. Although the formation of visible ribonuclear foci is still a hallmark of pathogenesis, more, and more evidence is indicating that the initial emergence of foci does not equal to immediate detectable toxicity. The foci do not initiate the disease process *per se*. In fact, the formation of foci could possibly be a healthy response to temporarily sequester the toxic RNA. Although in the long run, foci may still contribute to the progression of the disease by sequestering RNA-binding proteins, and gradually lead to degeneration.

Apart from the traditional advantages in genetics, the recent advances in CRISPR/Cas9 system and bioinformatics have become a powerful driving force that will surely aid *Drosophila* research to ascend to the next level. For instance, Liu and colleagues have developed an improved RNA injection-based CRISPR/Cas9 system that is highly efficient for creating desired mutagenesis in the *Drosophila* genome with a mutagenesis induction rate up to 88% (Bassett et al., 2013). O'Connor-Giles and colleagues have expanded the system through homology-directed repair to facilitate complex genome engineering, enabling the insertion of sizable DNA sequences to create targeted knock-ins and conditional knockouts (Gratz et al., 2014). The CRISPR/Cas9 system can also be used for tissue-specific tagging of endogenous proteins (T-STEP; Koles et al., 2015). In contrast to the GAL4/UAS overexpression system, which sometimes may change the localization patterns of proteins, T-STEP retains the endogenous protein localization patterns. Furthermore, it achieves tissue-specific labeling, allowing researcher to dissect tissue-specific functions of the protein of interest. Next generation sequencing is now widely available, and the access of disease information is also becoming more convenient for *Drosophila* geneticists as well. Online databases such as FlyBase has even introduced “Human Disease Model Reports” recently, further facilitating the studies of human diseases using the fruit fly (Millburn et al., 2016). With the development of these techniques and databases, we expect many new and improved fly models of human diseases to emerge soon.

In conclusion, *Drosophila* is a useful *in vivo* model system that can reveal novel mechanistic insights into human disorders, providing the foundation for translational research and therapeutic development. Looking into the future, we anticipate the tiny fruit fly to continue playing an important role in gene and drug discoveries in the ongoing battle between humans and neurodegenerative and neuromuscular diseases.

## Author contributions

ACK wrote the manuscript. HYEC contributed to the writing of the manuscript.

### Conflict of interest statement

The authors declare that the research was conducted in the absence of any commercial or financial relationships that could be construed as a potential conflict of interest.
